# Oral Squamous Cell Carcinoma: Features and Medico-Legal Implications of Diagnostic Omission

**DOI:** 10.1155/2024/2578271

**Published:** 2024-09-21

**Authors:** Davide Albano, Antonina Argo, Giuseppa Bilello, Enzo Cumbo, Melania Lupatelli, Pietro Messina, Fabio Massimo Sciarra, Mario Sessa, Stefania Zerbo, Giuseppe Alessandro Scardina

**Affiliations:** ^1^ Department of Promotion of Health, Maternal-child Internal Medicine and Specialist of Excellence “G. D'Alessandro” University of Palermo 90127, Palermo, Italy; ^2^ University of Palermo Department of Precision Medicine in Medical Surgical and Critical Care (Me.Pre.C.C.), Palermo, Italy; ^3^ Independent Researcher, Italy

**Keywords:** diagnostic delay, diagnostic omission, litigation, malpractice, medico-legal issues, oral squamous cell carcinoma (OSCC)

## Abstract

Early diagnosis of oncologic pathologies has a crucial role to determine patient's prognosis and therapeutic path. Nonetheless, clinical errors and omissions that can occur during diagnostic, as well as detection of preneoplastic or neoplastic condition, may result in devasting consequences both for patients in terms of health and for professionals in terms of medico-legal responsibility. This study is aimed at examining in depth, through the presentation of a specific clinical case, the medico-legal aspects inherent to the diagnosis of oral cancer, analyzing the preventive, interceptive, and diagnostic strategies, the legal implications of clinical evaluation errors and diagnostic omission, and the type of medical damage produced and professional liability. The medico-legal landscape surrounding oral squamous cell carcinoma is multifaceted and characterized by diagnostic challenges, treatment complexities, and legal considerations. Health-care providers must remain vigilant in navigating these complexities to ensure optimal patient care while mitigating legal risks. By prioritizing high-quality medical records, fostering transparent communication with patients, and implementing preventive strategies, health-care institutions can strive to minimize the incidence of litigation and uphold standards of ethical practice in oral carcinoma cases. Additionally, continued research and education in forensic and legal medicine are essential in informing evidence-based practices and promoting patient safety in this evolving field.

## 1. Introduction

Oral squamous cell carcinoma (OSCC) accounts for about 90% of malignant neoplasms of the oral cavity. Globally, oral cancer (including the lips and oral cavity) ranks as the 16th most common malignancy, with an incidence of about 350,000 new cases annually [1]. The 5-year survival rate is below 40% for diagnoses made at Stages III and IV, but it reaches up to 80% when the cancer is detected at an early stage (Stages I and II) [2]. Treatment of early-stage oral cancer patients suggests a better prognosis [3]. Unfortunately, there are still many new diagnoses (about 70%) at a late stage: it emphasizes the critical need for early detection and prevention strategies [4]. Early diagnosis of oral cancer is crucial to improve survival of the patient and reduce mortality, while simultaneously, early detection often leads to better treatment outcomes, not having to resort to invasive surgical therapies [5].

One of the most critical aspects of dental practice concerns diagnostic and treatment of oral pathology, especially when a severe disease as oral cancer is involved. Early diagnosis of oncologic pathologies has a crucial role to determine patient's prognosis and therapeutic path [2]. Nonetheless, clinical errors and omissions that can occur during diagnostic, as well as detection of preneoplastic or neoplastic condition, may result in devasting consequences both for patients in terms of health and for professionals in terms of medico-legal responsibility [6].

In the field of medical practice, the intersection between medicine and law plays a fundamental role in determining responsibilities, evaluating care, and defining professional standards. The medico-legal aspects and implications, therefore, represent a complex and critical domain in which health and justice intertwine. This branch of medicine encompasses a wide range of issues, including professional's liability, potential malpractice conduct, and adherence to standards of care [7].

The diagnostic omission, particularly in the context of OSCC, raises extremely important medico-legal questions: not only the failure to promptly identify this type of cancer can compromise the patient's survival prospects but it can also generate significant disputes and legal controversies regarding the actions and omissions of the dental and medical professionals involved [4, 5].

This study is aimed at examining, in depth, through the presentation of a specific clinical case, the medico-legal aspects inherent to the diagnosis of oral cancer, analyzing the preventive, interceptive, and diagnostic strategies, the legal implications of clinical evaluation errors and diagnostic omission, and the type of medical damage produced and professional liability. Exploring the legal implications associated with delayed diagnosis or misassessment of oral carcinoma is essential to understand the challenges and responsibilities in both medical and legal fields. Through this study, we aim to provide a clear view of the interconnected dynamics between medicine and law in the context of OSCC, highlighting the importance of a medical practice that should be timely and accurate, as well as suitable to professional and legal standards.

## 2. Case Presentation

**Figure 1:**
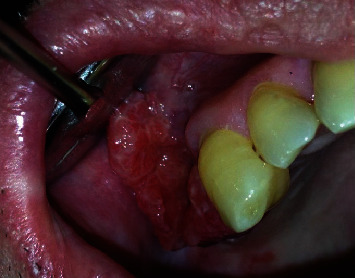
Type and localization of the oral lesion.

The case involves a 71-year-old man, nonsmoker, and in good general health, who complained of an enlarged gingival lesion at the right maxillary, persisting for at least 1 month. Oral examination revealed a hard nontender red mass characterized by an ulcerated surface, in adherent gingiva distally to tooth 1.3 and involving both the buccal and palatal side (Figure 1).

The lesion did not bleed upon palpation, and there was no palpable locoregional lymphadenopathy. Two months before the onset of the gingival mass, the patient was referred to his dental practitioner to extract teeth 1.4 and 1.5 due to significant mobility. In the weeks following the extraction, the skeletal prosthetic devices, made to compensate for the missing teeth in the first quadrant, were adjusted several times because the patient reported increasing discomfort and pain due to the pressure exerted by the prosthesis on the tissues of the previously treated area. Due to the rapid onset and the clinical features of the lesion and in the light of the potential presence of a malignant disease, an incisional biopsy of the lesion was instantly performed.

**Figure 2:**
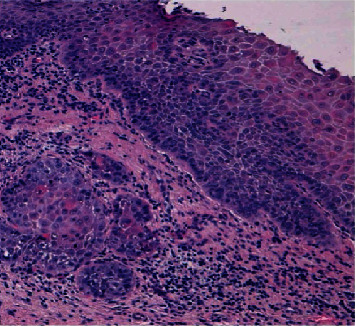
Moderately differentiated squamous cell carcinomas: islands of neoplastic atypical epithelial cells, oval-shaped, and round, which infiltrated the tumoral stroma. Pleomorphic, discohesive, and dyskeratotic tumor cells. Neoplastic islands were surrounded by fibrous stromal and inflammatory cells.

**Figure 3:**
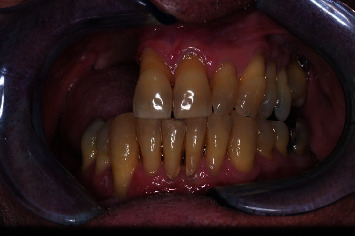
Patient's oral situation after partial maxillectomy surgical procedure.

**Figure 4:**
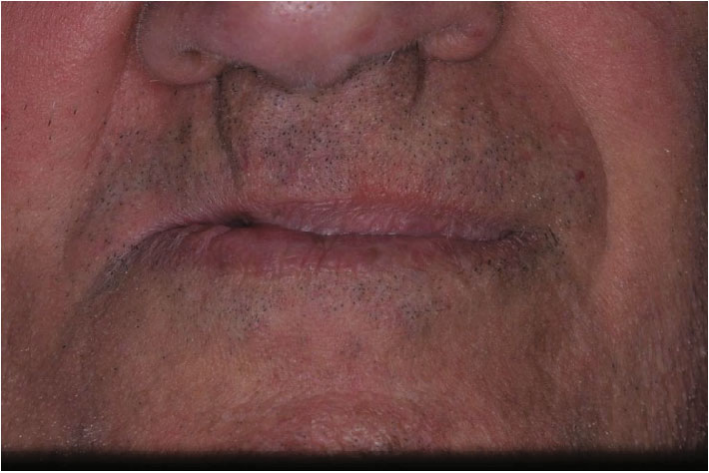
Right fornix reduction after partial maxillectomy surgical procedure.

Hematoxylin and eosin sections showed an infiltration of moderately differentiated squamous cell carcinoma (Figure 2). The patient was referred to the Department of Maxillofacial Surgery for staging and surgical treatment. A maxillectomy was performed and maxillary defect was reconstructed using a buccal fat pad and partial sliding local flaps. The oncological follow-up shows clearly visible postsurgical scarring and a significant reduction of the right fornix. This situation has led to masticatory difficulties for the patient. (Figures 3 and 4).

## 3. Discussion

According to the GLOBACAN 2020 data, OSCC annual mortality rate was 177,575 deaths. The mortality rate from this neoplasm still remains high in Italy as in other countries in the rest of the world, despite medical advances [1]. The risk of developing advanced cancer is high when there is a delay in diagnosis [8]. To summarize, diagnostic delay can primarily be linked to two key factors: patient-related delay (patient delay) and health-care professional–related delay (professional delay) [9]. Regarding OSCC and patient delay, the literature suggests that this is one of the primary reasons for diagnostic delay because patients are usually unaware of the symptoms of oral cancer and tend to associate them with dental or periodontal issues, thereby delaying the decision to consult a health-care professional [10]. On the other hand, professional delay is often associated with the lack of cultural and experiential background of the health-care professionals in promptly recognizing the symptoms and signs of such disease [2]. Preventive measures can reduce the risk of the disease developing. Disease prevention begins with educating the population about the signs and symptoms of diseases, understanding risk factors, discouraging the use of tobacco and the abuse of alcohol, and emphasizing the importance of regular check-ups and screenings [11]. About risk factor for OSCC, they are well-known. The major risk factors for OSCC include tobacco use in various forms, alcohol consumption, and betel nut chewing [12]. In recent decades, there has also been an increase in cases of oropharyngeal carcinoma associated with HPV (mainly HPV type 16), particularly among young patients, an age group that is generally less represented in squamous cell carcinoma incidences traditionally linked to the consumption of tobacco and alcohol [13, 14]. Ultraviolet radiation (UV) is also recognized as a risk factor in the pathogenesis of lip cancer [15]. Moreover, OSCC may be preceded by and arise from premalignant phases that are classified as potentially malignant oral disorders (OPMDs), which encompass a varied group of conditions with a heightened risk for oral cancer development [16]. The relatively predictable nature of OSCC has prompted researchers to develop screening strategies capable of detecting both patients with overt oral cancer and those with OPMDs as early as possible. The Visual-Oral Exam (VOE) is an opportunistic oral screening procedure extensively used in a multitude of studies and programs. It should be conducted during any routine visit, irrespective of the patient's reason for consulting an oral health specialist, and is aimed at analyzing perioral and all oral region tissues through inspection and palpation. The intraoral analysis relies on both direct and indirect visual examination of the oral mucosa, looking for tissue changes in color, volume, and texture. It is essential to underline that the oral screening process is noninvasive and it is painless. However, its significance in detection of OSCC or/and OPMDs cannot be underestimated. Subsequent to this procedure, the patient is referred to a specialist [17]. However, it is worth remembering that a screening test is not a diagnostic tool; the gold standard for the diagnosis of OSCC and OPMDs remains tissue biopsy and histopathological assays [18]. In the earliest stages, the tumor might be completely asymptomatic but still present as a visible alteration of the normal consistency and appearance of the mucosa [17]. A dental practitioner should be able to recognize a range of signs and symptoms during an oral cancer screening exam. Generally, the most common mucosal signs and symptoms are as follows: persistent ulcerative lesions; persistent white or red patch or plaque, or mixed (white and red); nodule or swelling; and localized area of bleeding.

Additionally, general dentists must be skilled in identifying any suspicious lesions, such as presumed trauma ulcers, that do not improve or persist for more than 2 weeks after the removal of potential local irritants and should refer these cases to a specialist for further evaluation [5].

The early stages of oral carcinoma often show nonspecific symptoms, leading to frequent delays in diagnosis and treatment approach. Diagnostic delay may lead to neoplastic progression. Furthermore, misinterpreting clinical findings or imaging studies can result in diagnostic errors, potentially leading to adverse patient outcomes. Radiographic interpretation, biopsy techniques, and histopathological analysis are areas prone to mistakes, highlighting the need for meticulous attention to detail and adherence to best practices [19].

Oral cancer presents a worldwide incidence of 377,713 new cases and 177,757 deaths per year. In patients with OSCC, delays in diagnosis of more than 1 month may contribute to an increased chance of diagnosis of later-stage disease [4, 15].

González-Moles, Aguilar-Ruiz, and Ramos-García demonstrated that the 5-year mortality rate for OSCC is close to 50%. Approximately 50% of OSCCs are diagnosed at an advanced tumor stage (T3/T4) and 47% of these advanced-stage diagnoses also present with a positive nodal status (N+). About 30% of oral cancer patients delay seeking help for more than 3 months after first noticing signs and symptoms. Survival of patients treated within the first month of symptom onset is 86% at 5 years, it drops to 47% if the diagnosis is made within 7 months of symptom onset, and after 12 months, the chances of survival are very poor. A significant association between late diagnosis and advanced tumor stages was reported, with late-diagnosed oral carcinomas being 30% more likely to be diagnosed at an advanced stage [15].

Dentists may identify HNC at an earlier stage than physicians. A survey of 51 new patients with OSCC showed that detection by dental-care providers during a routine examination was associated with a less advanced stage of cancer at diagnosis [4].

Patton and colleagues conducted a survey of medical- and dental-care providers in North Carolina regarding the adequacy of training received in the detection of OSCC. Dental-care providers felt adequately trained to conduct oral examinations, but they were less confident about conducting lymph node examinations than were medical-care providers [20].

Jones and colleagues reported a mean delay from general practitioners' referrals to specialists of 5.1 weeks, a mean delay in performing radiographical examinations of 5.6 weeks, and a mean time to treatment after referral of 10.3 weeks for radiation therapy and 5.2 weeks for surgery [18]. Anyway, *delays over 40 days* in treatment are associated with worse outcomes [4, 15].

The diagnosis and management of oral carcinoma are characterized by several medico-legal issues, particularly in the context of potential malpractice and litigation. Several factors contribute to the complexity of these cases, including diagnostic challenges, diagnostic delay, treatment complications, a significant impact on daily living, and informed consent–related issues. The adherence to guidelines and good clinical practices recommendations needs to be proven adequately. Indeed, the quality of medical records has a leading role in preventing litigation [21].

Wong and colleagues analyzed medical malpractice litigation related to oral cavity cancer from 2000 to 2019, evaluating a total of 65 lawsuits where with failure to diagnose being the most common allegation. *Diagnostic failure* was the most frequent allegation throughout both decades studied (58.8% in 2000–2010 and 74.2% in 2011–2019), followed by failure to biopsy (8.8%–32.3%), and failure to refer (8.8%–35.5%) from 2000–2010 to 2011–2019. Medical malpractice experiences were linked to increased physician burnout, highlighting the personal and professional toll of litigation on health-care providers [14].

Several studies have highlighted the significant role of medical records quality in determining medical care processes, risk management, and liability prevention [22]. A thorough documentation of medical records is indispensable for patient's care, as it is closely linked with patient medical outcomes. In addition, it reflects a proper organization of the health-care system. On the contrary, inadequate clinical documentation may compromise patient's safety and increase the risk of malpractice [23].

In 2007, the World Health Organization (WHO) promoted guidelines for medical records and clinical documentation, emphasizing its role in enhancing the health-care system quality. According to these guidelines, medical records must be clear, concise, correct, comprehensive, and contemporary, demonstrating that clinicians fulfilled their duty of care without compromising patient safety. Medical records and clinical documentation should include all aspects of patient care and serve as a repository for dental professionals' knowledge, observations, actions, decisions, and outcomes to facilitate a personalized approach [22, 24].

Thus, as we previously mentioned, medical records are crucial in evaluating dental professionals' liability in malpractice litigation. In judicial proceedings, the appropriateness of health-care practices is typically measured against accepted standards, often relying on guidelines or best clinical practices.

Dental professionals should be encouraged to practice evidence-based medicine, including accurate and guideline-based recording of medical records, in order to ensure patients' safety and prevent potential situations of legal disputes. The accurate and thorough collection of clinical documentation of preneoplastic lesions and oral carcinoma is essential. Besides, a proper management of every step of patient's therapeutic path (diagnosis, clinical and surgical treatment, radiotherapy, and follow-up) is crucial to minimize the possibility of litigation [25]. High-quality medical records are pivotal in medico-legal proceedings related to oral carcinoma cases. Comprehensive documentation of clinical encounters, diagnostic evaluations, treatment plans, and informed consent discussions facilitates the continuity of care and serves as invaluable evidence in legal defense. Complete and accurate medical records can help health-care providers in demonstrating their adherence to standard practices, refuting allegations of negligence and, obviously, safeguarding patient's well-being [19, 26].

The informed consent serves as a cornerstone of patient autonomy and ethical medical practice [27]. In the context of oral carcinoma, obtaining a proper informed consent implicates discussing the nature of the disease, treatment options, potential risks and benefits, and alternative approaches. Failure to adequately inform patients about their diagnosis or treatment alternatives can result in allegations of medical negligence and subsequent litigation. Therefore, a clear and complete documentation is crucial for the professional's defense against malpractice claims [28].

Patients diagnosed with OSCC place significant value on clear and empathetic communication from their health-care providers. They prioritize a thorough understanding of their diagnosis, treatment options, and potential outcomes through accessible language and visual aids. Informed consent holds particular importance, not only merely signifying procedural compliance, but also a comprehensive understanding of treatment risks and benefits and alternative therapies. Enhancing the doctor–patient relationship in oral cancer care necessitates active listening, validation of patient concerns, and collaborative decision-making. Central to this approach is the respect for patient autonomy, enabling individuals to make informed decisions congruent with their values and preferences [29, 30].

However, treatment-related complications, such as surgical errors, radiation-induced mucositis, or chemotherapy toxicity can occur, sometimes resulting in patient's injury. In such instances, features as the appropriate gathering of the informed consent, a well-detailed documentation of treatment decisions, and the adherence to the established protocols are essential in mitigating legal risks [31].

The medical equipment of the multidisciplinary group (dentists, oral pathologists, oncologists, surgeons, etc.) must provide patients with oral cancer or suspected preneoplastic or neoplastic lesions comprehensive information about multiple issues. Patients must be informed about preventing oral cancer through regular clinical examinations and avoiding harmful habits. Treatment options for oral cancer depend on factors such as cancer type and stage, with surgery being the most common approach. The patient's refusal to undergo oral cancer treatment exempts the professional from liability, even if the patient is informed about potential risks of forgoing the proposed treatment [32].

As previously mentioned, the management of oral carcinoma typically involves a multimodal approach, including surgery, radiation therapy, chemotherapy, or a combination of both. Anyway, a biopsy is essential for a proper diagnosis of suspected malignant lesions and the subsequent treatment planning [31, 33].

Patients should be informed about the potential acute and long-term complications of surgery, radiotherapy, and chemotherapy, including mucositis, dysgeusia, xerostomia, infectious diseases, pain, cranial nerve damage, abscesses, and difficulties with chewing and swallowing. They should also receive a proper guidance on maintaining a proper oral hygiene, nutrition, and lifestyle habits during treatment, in order to minimize general complications [32, 34].

The management of complications requires a thorough assessment of the situation. It is indispensable to identify the type and extent of injury to determine the most effective intervention strategy, as well as to know appropriate protocols to deal promptly with adverse events and critical or unforeseen situations [35].

As outlined above, a proper informed consent should summarize the verbal information provided to the patient, tailored to their educational level, comprehension skills, and emotional status, allowing them to accept or refuse treatment [28, 32].

The risk management is a crucial aspect to ensure the patient's safety and quality of provided therapy.

When clinical risk situations are detected, a suitable collection of documentation can certify the information provided and emphasize the need for regular check-ups and risk factor reduction. Furthermore, in cases of suspected oral lesions, clinical records should clearly outline the need for diagnostic investigations, potential therapies, and possible complications [36].

The collection of documentation in patients with advanced oral cancer should carefully summarize all the information regarding the purposed treatment and associated acute and long-term complications. Patients' right to refuse specific treatments should be respected and documented in the clinic paperwork, including customizable sections and space for additional notes [31].

When medical complications occur, it will be essential to inform the patient clearly and openly about the situation: transparency in reporting adverse events occurred and actively involving the patient in the decision-making process can both help maintain the doctor–patient trust relationship and manage the complications in the best possible way [36].

Litigation for alleged diagnostic omission or medical malpractice in oral carcinoma cases can have far-reaching economic consequences for patients, professionals, and health-care systems [6, 37].

OSCC late diagnoses often leads to more advanced stages of diseases, necessitating intensive treatments and care. The economic impact results in higher health-care costs, compared to early detection where treatments are less invasive and less costly. Moreover, late diagnoses can sometimes result in litigation due to perceived medical negligence or malpractice. Subsequently, legal battles incur costs for both health-care providers and patients, including legal fees, settlements, and potential damages. On the contrary, early detection of OSCC allows cost-savings in healthcare, reduced legal risks and, as a consequence, overall enhancement of quality of life for individuals and society [38].

In legal medicine, the concept of “loss of chance damage” stands as a stark reminder of the profound consequences that diagnostic omissions can exact, particularly in the realm of oral carcinoma. With each instance of overlooked or delayed diagnosis, a crucial window of opportunity is shuttered, leaving behind missed chances and possible irreparable harm. It can occur when the opportunity for timely intervention is squandered. In the context of oral carcinoma, this manifests as the forfeiture of the chance to intercept the disease at its nascent stage, when treatment modalities wield their maximal efficacy and prognosis remains relatively favorable [39].

OSCC poses an arduous challenge due to its often-insidious onset and subtle early symptoms. The delicate balance between benign oral lesions and malignant counterparts underscores the critical role of timely and accurate diagnosis in altering the course of disease progression [2, 5].

The ramifications of loss of chance damage are multifaceted, extending far beyond the realm of clinical outcomes. At its core lies the individual patient, whose journey is irrevocably altered by the missed opportunity for early diagnosis [38]. What could have been a scenario characterized by timely intervention and hopeful prognosis devolves into uncertain clinical predictions, as cancer proliferates and metastasizing and diminishing the prospects of meaningful recovery [19].

From a broader perspective, the societal burden of loss of chance damage weighs heavily on health-care systems already grappling with finite resources and competing priorities. The economic toll of advanced-stage presentations, necessitating intensive treatments and palliative care measures, further underscores the imperative of early detection in mitigating health-care expenditure and optimizing resource allocation [40].

The costs associated with defending malpractice claims, including legal fees, expert witness fees, and potential settlement payouts, can be substantial. Furthermore, litigation-related stress and reputational damage may impact health-care providers' professional well-being and patient trust [41, 42]. From a societal perspective, the economic burden of litigation can strain health-care systems and contribute in the health-care costs rise, underscoring the importance of proactive risk management and litigation prevention efforts [43, 44].

Lack of informed consent is present in many medical malpractice claims [45]. Furthermore, communication deficiencies have been identified as a critical factor in malpractice claims. Patient's involvement in medical decisions decreases the number of malpractice claims related to medical assistance [46, 47]. Undoubtedly, an appropriate oral and written communication will ensure a key role during the therapeutic path, in order to promote an advantageous professional–patient human relationship and to manage adequately the clinical risk [41].

## 4. Conclusions

As of today, the only truly effective method for the early diagnosis of squamous cell carcinoma is the routine assessment of a physical examination on all patients, regardless of their reason for consulting the specialist. Furthermore, general dentists must be proficient in identifying and refer to a specialist for further evaluation any suspicious lesion (such as estimated trauma ulcers) that shows no signs of improvement or that persists after an average period of 2 weeks following the removal of potential local irritants.

The medico-legal landscape surrounding OSCC is multifaceted and characterized by diagnostic challenges, treatment complexities, and legal considerations. Health-care providers must remain vigilant in navigating these complexities to ensure optimal patient care while mitigating legal risks. By prioritizing high-quality medical records, fostering transparent communication with patients, and implementing preventive strategies, health-care institutions can strive to minimize the incidence of litigation and uphold standards of ethical practice in oral carcinoma cases. Additionally, continued research and education in forensic and legal medicine are essential in informing evidence-based practices and promoting patient safety in this evolving field.

## Data Availability

All data related to the presented manuscript are included within the article.
